# Stimulation of Insect Herbivory by Elevated Temperature Outweighs Protection by the Jasmonate Pathway

**DOI:** 10.3390/plants9020172

**Published:** 2020-02-01

**Authors:** Nathan E. Havko, George Kapali, Michael R. Das, Gregg A. Howe

**Affiliations:** 1 Department of Energy-Plant Research Laboratory, Michigan State University, East Lansing, MI 48824, USA; havkonat@msu.edu (N.E.H.); kapal2@uic.edu (G.K.); dasmicha@mit.edu (M.R.D.); 2 Plant Resilience Institute, Michigan State University, East Lansing, MI 48824, USA; 3 Department of Biochemistry and Molecular Biology, Michigan State University, East Lansing, MI 48824, USA

**Keywords:** jasmonate, elevated temperature, climate change, *Trichoplusia ni*, plant-insect interaction, Arabidopsis

## Abstract

Rising global temperatures are associated with increases in the geographic range, population size, and feeding voracity of insect herbivores. Although it is well established that the plant hormone jasmonate (JA) promotes durable resistance to many ectothermic herbivores, little is known about how JA-mediated defense is influenced by rising temperatures. Here, we used the Arabidopsis-*Trichoplusia ni* (cabbage looper) interaction to investigate the relative contribution of JA and elevated temperature to host resistance. Video monitoring of *T. ni* larval behavior showed that elevated temperature greatly enhanced defoliation by increasing the bite rate and total time spent feeding, whereas loss of resistance in a JA-deficient mutant did not strongly affect these behaviors. The acceleration of insect feeding at elevated temperature was not attributed to decreases in wound-induced JA biosynthesis, expression of JA-responsive genes, or the accumulation of defensive glucosinolates prior to insect challenge. Quantitative proteomic analysis of insect frass, however, provided evidence for a temperature-dependent increase in the production of *T. ni* digestive enzymes. Our results demonstrate that temperature-driven stimulation of *T. ni* feeding outweighs the protective effects of JA-mediated resistance in Arabidopsis, thus highlighting a potential threat to plant resilience in a warming world.

## 1. Introduction

In the coming decades, global climate change is expected to increase the severity of multiple stresses on plants and other members of terrestrial ecosystems [1,2]. In addition to higher average temperatures, increases in the frequency and severity of extreme weather events, including heat waves, floods, and drought, are anticipated [3,4,5]. As plants are subjected to continuous changes in abiotic conditions, so too are the pests and pathogens that consume plants as a source of nutrition. Increasing evidence indicates that climate change is altering the geographical range, population size, and feeding behavior of insect herbivores [6,7,8]. Modeling studies further predict that temperature-driven increases in insect metabolism will be a major factor contributing to crop losses in a warming world [6]. The rate at which insect herbivores consume food depends not only on the metabolic needs of the herbivore but also on the quality of host tissue and efficacy of defenses deployed by the plant host [9,10]. Because both plants and phytophagous insects are ectothermic organisms, it is critical to disentangle the influence of temperature on plant defense pathways from the physiological effects of temperature on the herbivore. These considerations provide a compelling rationale for research aimed at understanding how warming temperatures impact the highly evolved relationships between plants and arthropod herbivores.

Plant survival under combined biotic and abiotic stress depends on complex signaling systems that perceive threats and prioritize protective responses. Mitigation of high temperature stress relies on thermosensory systems to control a suite of acclimatory responses [11]. One such signaling system uses phytochrome B (phyB) to sense changes in light quality and temperature to alter shoot architecture for shade avoidance and evaporative leaf cooling responses, respectively [12,13,14]. Upon perceiving biotic threats, plants rely on the jasmonate (JA) signaling pathway to activate the expression of multi-layered defensive traits that curtail herbivory [15]. Mechanical wounding and herbivory generate signals that propagate through the plant to activate the biosynthesis of JA from plastidial galactolipids [16,17,18]. Tissue damage triggers enzymatic oxidation of linolenic acid via the plastidic lipoxygenase/allene oxide synthase/cyclase (LOX/AOS/AOC) cascade to generate the cyclic oxylipin 12-oxo-phytodienoic acid, which is subsequently converted to jasmonic acid and jasmonoyl-L-isoleucine (JA-Ile) in the peroxisome and cytosol, respectively [18]. In the nucleus, JA-Ile promotes the assembly of a co-receptor complex in which the F-box protein CORONATINE INSENSITIVE1 (COI1) contacts JASMONATE ZIM DOMAIN (JAZ) transcriptional repressor proteins, leading to JAZ ubiquitination and degradation [19,20,21]. Transcription factor targets of JAZ are thereby released from repression to activate the expression of defense-associated genes [22,23,24,25].

Recent studies have begun to unravel the complex effects of elevated temperature on molecular pathways that govern plant–microbe interactions [26]. Work with *Arabidopsis thaliana* (Arabidopsis), for example, has shown that moderate increases in ambient temperature impair the biosynthesis of the defense hormone salicylic acid (SA), thereby compromising resistance to the bacterial pathogen *Pseudomonas syringae* [27]. Considerably less is known about how increased temperature influences plant resistance to arthropod herbivores, which is mediated mainly by the JA signaling pathway [28,29,30]. Although it is well established that rising temperatures increase the feeding activity of certain ectothermic herbivores [6,31], the extent to which this effect involves perturbation of the JA pathway remains to be addressed.

Here, we investigated this question by studying the interaction of Arabidopsis with the generalist herbivore *Trichoplusia ni* (cabbage looper), a highly dispersive pest found in all geographic and climate regions where crucifers are cultivated [32]. To simulate a mild heatwave event, plants were allowed to develop under ambient temperature (22 °C) and then, were either maintained at that temperature or were shifted to a moderately elevated (29 °C) temperature prior to challenge with *T. ni* larvae. Video monitoring of *T. ni* behavior revealed that elevated temperature dramatically stimulated defoliation by accelerating the bite rate and increasing the proportion of time spent feeding. We also found that the increased rate of insect feeding was not attributed to a defect in wound-induced JA accumulation or reduced accumulation of defensive glucosinolates prior to insect challenge. Experiments involving JA-deficient mutants of Arabidopsis further showed that although the JA pathway confers some resistance to herbivory at the warmer temperature, this level of protection was overwhelmed by the powerful stimulatory effect of elevated temperature on insect feeding. Elevated temperature promoted faster larval growth on defended wild-type plants compared with larval growth on defenseless *aos* mutants under control conditions. Proteomic analysis of insect frass (feces) suggested that elevated temperature alters digestive physiology to accommodate increased leaf consumption. Collectively, our findings suggest that temperature-driven changes in *T. ni* feeding behavior overpower native mechanisms of JA-induced resistance in Arabidopsis.

## 2. Results

### 2.1. Elevated Temperature Increases T. ni Feeding Activity on Wild-type and JA-deficient Arabidopsis Plants

**Figure 1:**
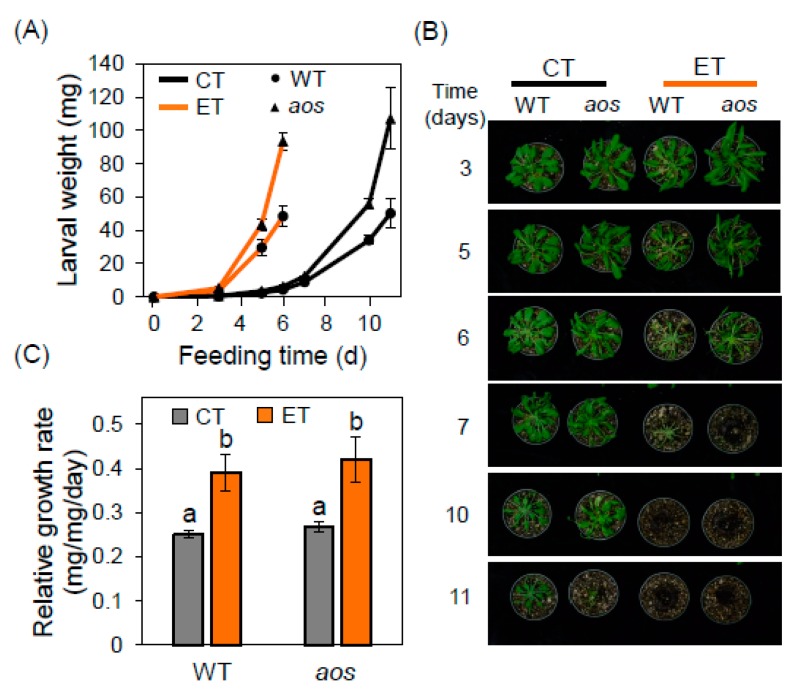
Elevated temperature powerfully stimulates *T. ni* growth and host plant defoliation. (**A**) Weight of *T. ni* larvae reared on rosettes of WT (circle) or *aos* (triangle) plants at the control temperature (CT, 22 °C; black lines) or elevated temperature (ET, 29 °C; orange lines). All plants were grown at CT for 8 weeks and then acclimated to ET or CT conditions for an additional 48 h prior to challenge with two neonate *T. ni* larvae per plant. Data points denote the mean ± SE of larvae recovered from five plants. (**B**) Representative photographs showing the defoliation of rosettes from plants used for feeding assays shown in panel A. (**C**) Relative growth rate (RGR) of larvae reared on WT or *aos* plants under CT (gray bars) and ET (orange bars) conditions. RGR was calculated from the data shown in panel A, where the data denotes the mean ± SE of five biological replicates. ANOVA tests indicated that temperature, but not genotype or genotype x temperature interaction, has a significant effect on RGR (P < 0.05). Lowercase letters denote significant differences (Tukey’s honestly significant difference [HSD] test P < 0.05).

To investigate the interaction between elevated temperature and JA-based resistance to insect herbivores, we challenged wild-type (WT) Arabidopsis and the JA-deficient allene oxide synthase (*aos*) mutant with larvae of the lepidopteran insect *T. ni* under control temperature (CT, 22 °C) and elevated temperature (ET, 29 °C) conditions. Plants grown under CT conditions for eight weeks were acclimated for 48 h to test temperatures (CT or ET) and then challenged with neonate larvae. Under CT conditions, larvae reared on WT plants for 11 days consumed the majority of leaf tissue and achieved a final weight of 50.2 ± 8.6 mg (Figure 1A,B). Insects reared on *aos* plants for the same period of time were heavier (107.0 ± 18.5 mg) than WT-reared larvae, consistent with previous studies [33]. Feeding trials performed under ET conditions showed that the shift from 22 °C to 29 °C greatly accelerated insect growth and leaf consumption on both WT and *aos* plants; these feeding assays were terminated after 6 days due to complete defoliation (Figure 1B). *aos*-reared larvae were ~two-fold heavier than WT-reared larvae under ET conditions, indicating that JA-based defenses are effective at the higher temperature (Figure 1A). Strikingly, however, the effect of temperature on *T. ni* performance was much greater than the effect of host genotype. For example, larval weight gain on WT plants after six days of feeding under ET conditions was 11-fold greater than that of larvae grown on WT plants under CT conditions for the same period of time (Figure 1A). Estimates of the relative growth rate (RGR) of larvae under the four experimental conditions (two genotypes x two temperatures) further supported the conclusion that the effect of temperature on insect weight gain was much greater than the effect of JA biosynthetic capacity (Figure 1C). Specifically, the RGR of larvae reared on WT plants at ET increased by 55% (from 0.25 ± 0.01 to 0.39 ± 0.04 mg/mg/day) relative to larvae on WT plants under CT conditions. In contrast, JA biosynthetic capacity had a minor effect on larval growth rate; the RGR of larvae on *aos* plants at CT trended toward an increase by 7% (to 0.27 mg/mg/day), which was not significant (P > 0.05) relative to insects on WT plants at CT. Under ET conditions, the RGR of larvae on *aos* plants (0.42 ± 0.05 mg/mg/day) showed a similar trend with a non-significant increase (P > 0.05) by about 8% compared to larvae on the WT host (Figure 1C).

### 2.2. Elevated Temperature Does Not Compromise Wound Responses or Bulk Glucosinolate Production

**Figure 2:**
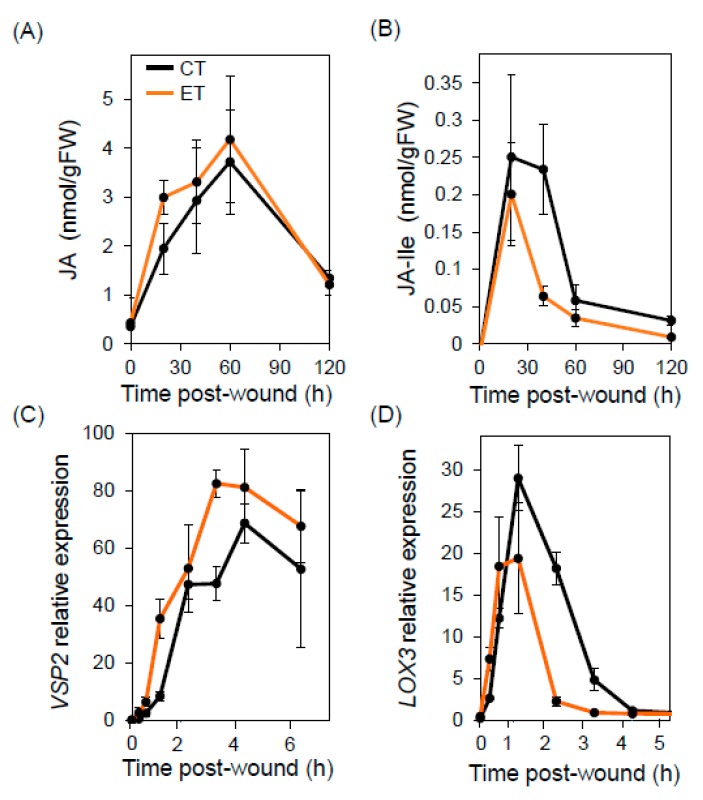
Elevated temperature conditions do not compromise wound responses. Plants were grown continuously at CT for 38 days prior to a 48-h acclimation period at either CT or ET. Following the acclimation period, three leaves of each plant were mechanically wounded with a hemostat. Wounded leaves were collected at the indicated times thereafter for analysis of hormone or gene expression levels. (**A**,**B**) Time course of wound-induced jasmonic acid (JA, **A**) and JA-Ile (**B**) accumulation. The black and orange lines denote plants grown continuously at CT (22 °C) and plants that were exposed to ET conditions (29 °C) for two days prior to wounding, respectively. (**C**,**D**) Wound induction of the JA-responsive genes *VSP2* (**C**) and *LOX3* (**D**) in plants grown as described above. The data points denote the mean ± SE of three replicates containing tissue pooled from two plants.

To test whether compromised JA responses contribute to the accelerated insect growth at ET, we measured wound-induced accumulation of the bioactive form of JA, jasmonoyl-L-isoleucine (JA-Ile), and its immediate precursor, jasmonic acid. Plants were grown under CT conditions for 38 days and then split into two groups for a 48-h acclimation period at either CT or ET. Mechanical wounding of leaves led to rapid and transient production of JA and JA-Ile, with peak levels observed approximately 60 and 15 min after wounding, respectively (Figure 2A,B). Maximal accumulation of JA and JA-Ile was similar under CT and ET conditions, indicating that the ET regime used in our experiments does not substantially alter the accumulation of bioactive JAs. Similarly, an analysis of gene expression by quantitative PCR showed that the wound-induced expression pattern of the JA-responsive marker genes *VEGETATIVE STORAGE PROTEIN 2* (*VSP2*) and *LIPOXYGENASE 3* (*LOX3*) was comparable under CT and ET conditions (Figure 2C,D). These data indicate that JA-dependent wound responses remain intact under the ET conditions used for insect feeding assays.

**Figure 3:**
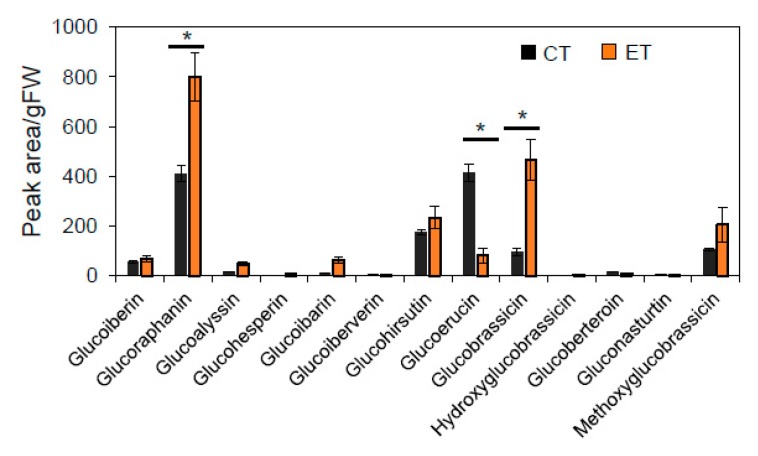
Elevated temperature alters the pattern of glucosinolate accumulation in Arabidopsis leaves. Plants were grown continuously at CT for four weeks prior to a 7-day acclimation period at either CT (black bars) or ET (orange bars), after which time leaves were sampled for measurement of glucosinolate content by LC-MS. The data show the abundance of glucosinolate species per gram fresh weight (g FW) leaf tissue obtained from three biological replicates. The asterisks denote significant differences between ET and CT according to Tukey’s HSD test (P < 0.05).

Resistance of Arabidopsis to *T. ni* is dependent on the production of toxic glucosinolate derivative produced upon activation of myrosinases in wounded tissue [34]. To test whether the increased rate of *T. ni* feeding at the higher temperature is associated with reduced glucosinolate levels, we used liquid chromatography-mass spectrometry (LC-MS) to measure the leaf glucosinolate content of plants grown for four weeks under CT conditions and then transferred to an ET treatment chamber for seven days (or maintained at CT). Among a total of 13 glucosinolate species detected in WT leaves, two compounds (glucoraphanin and glucobrassicin) accumulated to higher levels in plants that received the seven-day ET treatment compared to CT control plants (Figure 3). Only one compound, glucoerucin, showed decreased abundance in leaves of ET-treated plants. These data demonstrate that transient exposure of CT-grown plants to moderate heat stress conditions (29 °C for seven days) does not deplete the bulk glucosinolate content in undamaged leaves. Rather, this ET regime is associated with quantitative changes in the abundance of a subset of a glucosinolate species, with most of these compounds increasing in abundance at ET.

### 2.3. Elevated Temperature Alters Insect Feeding Behavior Independently of Jasmonate Defenses

**Figure 4:**
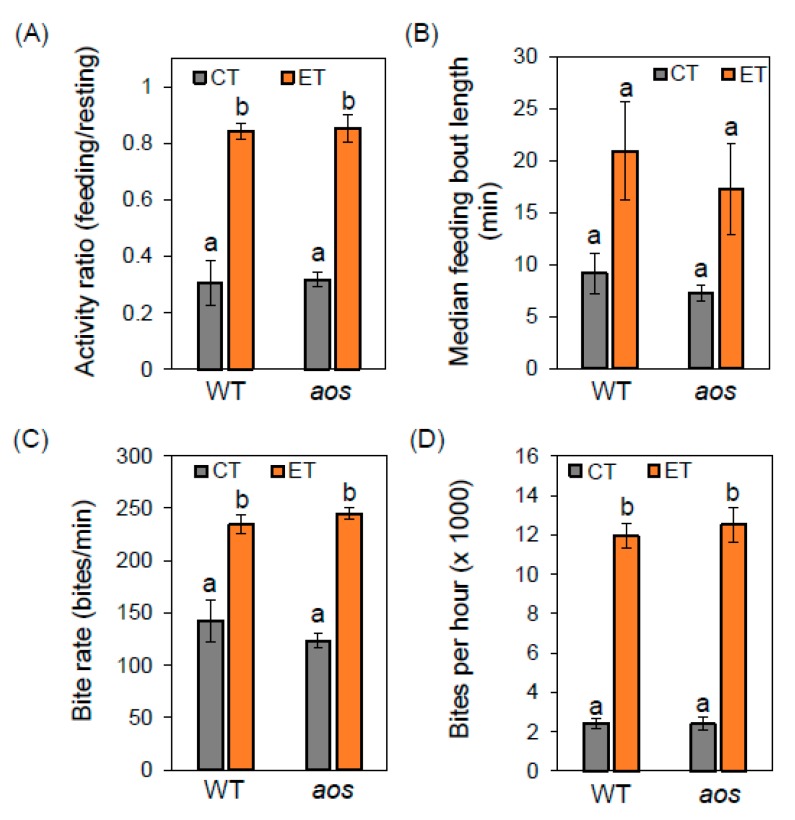
*T. ni* larvae spend more time feeding and feed faster at elevated temperature. WT and *aos* mutant plants were grown continuously at CT for eight weeks and then transferred to CT or ET treatment chambers. Following a 24-h acclimation period in the treatment chamber, plants were challenged with *T. ni* larvae that had previously been reared to the 3rd instar stage (~35 mg) on WT plants under CT conditions. Within the treatment chambers, larvae were allowed to feed for 24 h prior to monitoring of feeding behaviors by video recording for approximately seven hours. (**A**) The data show the ratio of time spent feeding to the time spent resting (i.e., not feeding) on the indicated host genotype under CT (grey bars) or ET (orange bars) conditions. (**B**) The average length of feeding bout for larvae feeding on the indicated plant genotype and temperature (CT, grey bars; ET, orange bars). (**C**) Bite rate during a feeding bout. Cameras were continuously adjusted to maintain focus on the movement of larval mandibles. Feeding rates were determined by sampling the average number of bite-motion cycles per minute. (**D**) The average number of bites per h was estimated by multiplying the average bite rate during continuous feeding (panel C) by the activity ratio estimates in panel A. The data represent the mean and SE of three biological replicates. Lowercase letters denote significant differences (Tukey’s honestly significant difference [HSD] test P < 0.05).

To investigate the influence of temperature and JA-mediated defenses on insect feeding behavior, we used real-time video monitoring to measure insect feeding activity. Larvae were reared on WT plants under CT conditions before transfer to test conditions (22 °C or 29 °C; WT or *aos* plants). Following a 24-h acclimation period at the test condition, feeding behavior was recorded for ~ seven hours during the light period of the light/dark cycle, beginning ~three hours after the dark-to-light transition. The videos were analyzed manually to generate time-dependent traces of feeding behaviors for individual larva (Video S1 and Figure S1). Tracing data from multiple insects were then combined to determine the average behavioral parameters. Under CT conditions, larvae on WT plants spent approximately 30% of the recorded time actively feeding, with very similar results obtained for larvae feeding on *aos* plants (Figure 4A). Under ET conditions, the total proportion of time spent feeding increased dramatically to approximately 80% on both WT and *aos* plants (Figure 4A). Consistent with this observation, the median length of feeding bouts (i.e., time between periods of non-activity) showed a consistent trend toward increased bout duration under ET conditions (but not statistically significant), with this effect being independent of host genotype (Figure 4B).

The high resolution of the camera used for these experiments allowed us to observe mandible movements of individual larvae, and to use the video recordings to estimate the average rate (bite rate) at which larvae removed leaf tissue in discrete bites. During continuous feeding at CT, the average bite rate was approximately 130 bites per minute for insects feeding on both WT and *aos* plants. At the higher temperature, however, the bite rate increased by nearly two-fold (∽230 bites per minute) on both host genotypes (Figure 4C). Based on observations of the bite rate and proportion of time spent feeding, we calculated that ET conditions increased the total number of bites taken per hour by about six-fold, with the host genotype having little or no effect on the overall rate of defoliation (Figure 4D).

### 2.4. Elevated Temperature Influences the Composition of Digestive Proteins

**Figure 5:**
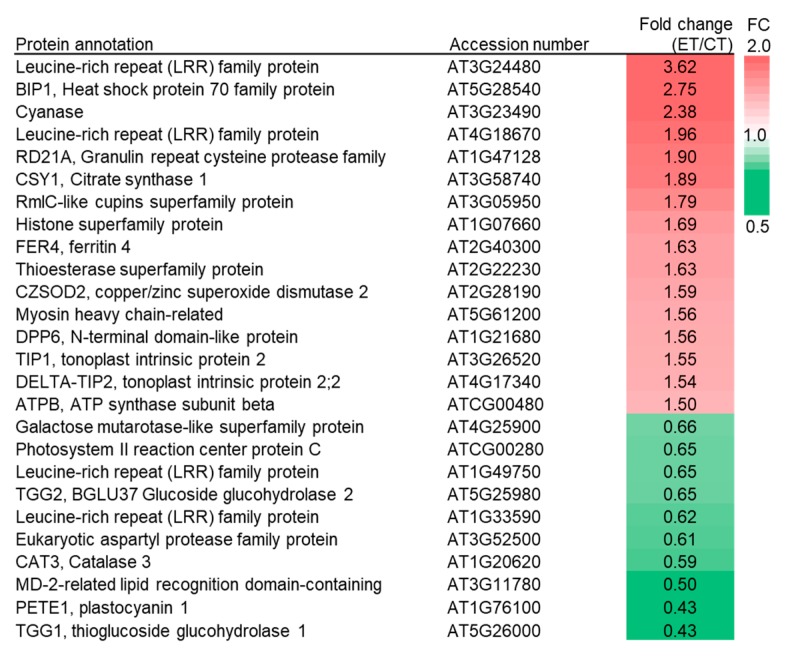
Differential accumulation of Arabidopsis proteins in frass from *T. ni* larvae reared at control versus elevated temperature. *T. ni* larvae were reared on WT Arabidopsis plants under CT or ET conditions as described in the main text. Arabidopsis proteins excreted in caterpillar frass were identified and quantified using a tandem-mass tag shotgun proteomics approach (see Methods). Listed are all Arabidopsis proteins showing increased (red) or decreased (green) abundance under ET compared to CT conditions (1.5-fold cutoff for ET/CT).

The strong effect of warmer temperature on *T. ni* feeding behavior raised the possibility that temperature influences digestive processes in the insect gut. To address this question, we used a tandem-mass tag shotgun proteomics approach [33,35] to quantify the abundance of both Arabidopsis (i.e., dietary) and *T. ni* proteins in frass (i.e., feces) collected from larvae feeding on WT plants at CT and ET. Several studies have suggested that plant proteins involved in anti-insect defense have evolved for increased stability in the herbivore gut; as a consequence, the presence of undigested plant proteins in frass may provide a measure of the efficiency of processes involved in food digestion [33,35]. Based on an analysis of six frass samples (biological triplicates for CT and ET treatments), a total of 136 unique Arabidopsis proteins were identified with high confidence in both CT and ET samples (Table S1). Using a 1.5-fold threshold as the criterion for differential accumulation, 16 Arabidopsis proteins accumulated to higher levels in frass from larvae feeding at ET relative to CT conditions, whereas 10 host proteins were less abundant under ET conditions (Figure 5). Consistently with our finding that higher temperature does not markedly alter the wound-induced level of JA/JA-Ile or defense-related transcripts (Figure 2), the list of differentially accumulating Arabidopsis proteins was not enriched in wound- or JA-responsive proteins (Figure 5). However, we did find that the TGG1 and TGG2 myrosinases implicated in glucosinolate-mediated resistance to *T. ni* [34] were less abundant in frass collected from ET-reared larvae relative to CT-reared larvae (Figure 5).

**Figure 6:**
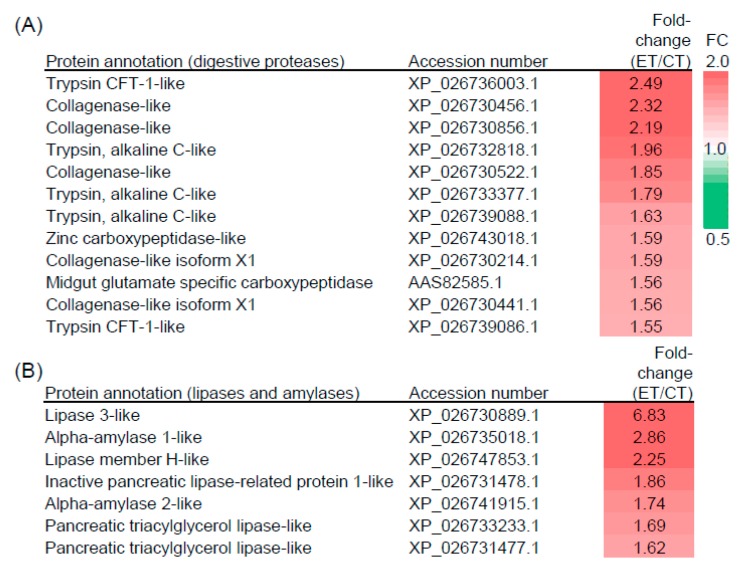
Feeding at elevated temperature increases the abundance of *T. ni* digestive enzymes. *T. ni* larvae were reared on WT Arabidopsis plants under CT or ET conditions as described in the main text. *T. ni* proteins excreted in caterpillar frass were identified and quantified using a tandem-mass tag shotgun proteomics approach. All the proteins listed have putative roles in proteolysis (**A**) or lipid/carbohydrate digestion (**B**) and are more abundant in frass from ET-reared compared to CT-reared larvae (1.5-fold change cutoff for ET/CT).

Searches of the frass protein mass spectral data against the *T. ni* genome sequence [36] identified 108 high-confidence *T. ni* proteins common to both the CT and ET treatments (Table S2). As expected for proteins excreted in frass, this list was highly enriched in putative digestive enzymes involved in the breakdown of leaf dietary proteins, lipids, and carbohydrates. Among the 37 proteins annotated as having a proteolytic-like function (Table S2), 12 accumulated to higher levels in frass from larvae that fed under ET conditions (Figure 6). Importantly, none of the 37 putative proteases were less abundant under ET conditions using the same 1.5-fold threshold for differential accumulations. A similar trend was observed for excreted *T. ni* proteins annotated as lipases or amylases. Specifically, we identified seven putative lipase or amylase enzymes that were more abundant in frass from ET-reared larvae compared to CT-reared larvae, whereas none of these proteins were less abundant in ET samples (Figure 6 and Table S2). These data indicate that the temperature-driven increase in the rate of leaf consumption by *T. ni* larvae is associated with elevated production of digestive enzymes in the insect gut.

## 3. Discussion

Despite predictions that rising temperatures and more frequent heatwaves will increase pressure on plants by increasing the metabolic demands and population size of insect herbivores [6,7], little is known about how this aspect of climate variability may affect specific plant anti-insect defense mechanisms, including induced resistance mediated by the JA pathway. Here, we addressed this question by assessing the impact of a moderate increase (7 °C) in temperature on well-characterized JA-dependent wound responses in Arabidopsis. Wound-induced JA accumulation, gene expression, and the production of defensive glucosinolates were largely unaffected by our ET treatment conditions. The use of the JA-deficient *aos* mutant in feeding assays permitted a comparison of the efficacy of JA-mediated resistance under CT and ET. The finding that larval weight gain increased on *aos* plants under both temperature regimes shows that JA-based resistance is operational at the higher temperature.

Remarkably, however, *T. ni* larvae grown on WT plants at ET gained much more weight than insects reared on the *aos* mutant at CT. In showing that elevated temperature can supersede the protective effects of JA, our findings highlight the importance of temperature as a dominant factor in determining the outcome of the *T. ni*-Arabidopsis interaction. Similar results were reported in a recent study that examined the effects of elevated temperature on the interaction between *Manduca sexta* (tobacco hornworm) and a JA perception mutant of cultivated tomato [37]. These collective findings suggest that projected increases in average global temperature [38] have the potential to disrupt plant–herbivore relationships that have co-evolved over the millennia and to tip the balance of these adversarial relationships toward the side of increased herbivory and greater plant damage.

Although we did not observe significant effects of ET on the capacity of Arabidopsis (a winter annual) to deploy JA-mediated defenses, it is possible that plants in warmer native environments have experienced selective pressure from temperature-stimulated insect outbreaks and therefore, possess temperature-sensitive mechanisms to enhance resistance to insect herbivores. This hypothesis is consistent with the variable effects of rising temperature on different insect herbivore–plant pairs [31], as well as the recent finding that temperature-dependent accumulation of HSP90 enhances JA-dependent wound responses by a mechanism likely involving COI1 stabilization [37]. Although ET conditions greatly stimulated the feeding activity of *T. ni* larvae, additional studies are needed to determine the effect of this climate variable on other stages of the insect life cycle [38,39,40]. Finally, we note that the recent discovery of phyB as a temperature sensor [11,12,13], together with the observation that loss of phyB relaxes growth-defense tradeoffs in a *jaz* quintuple mutant of Arabidopsis [41], opens new avenues for research to explore the complex effects of light quality and temperature on plant–insect relationships.

We investigated the effects of JA and temperature on the rates and patterns of *T. ni* feeding with real-time video imaging, using a camera system with sufficient resolution to discern mandible motions. This imaging system allowed us to quantify various insect feeding behaviors that contribute to rates of defoliation and larval growth. We found that the temperature shift from 22 °C to 29 °C increased the proportion of time spent actively feeding from about 30% to over 80%, and also increased the insect bite rate nearly two-fold. Interestingly, the absence of JA-based defenses in the *aos* mutant did not appreciably alter the total proportion of time spent feeding, the length of feeding bouts, or the rate of biting under either CT or ET conditions. Given that larvae feeding on *aos* plants grew faster than their WT-reared counterparts, we suggest that JA-mediated defenses impair the efficiency of post-ingestive processes that fuel larval growth from dietary (i.e., leaf) nutrients. It is also possible that slower larval growth on WT relative to *aos* plants reduces total consumption of host tissue because smaller larvae remove a smaller leaf area per bite. Once ingested, potential targets of Arabidopsis defense compounds include digestive enzymes and midgut epithelial cells involved in nutrient production and acquisition [42,43]. Delayed maturity of insect herbivores by host plant defenses can increase the risk of predation by organisms at the third trophic level [44]. Conversely, temperature-driven increases in the rate at which insect herbivores develop and mature may decrease the effectiveness of natural enemies [45], thereby compounding the negative effects of warmer temperatures on plant resilience.

To gain insight into the insect physiological processes associated with increased *T. ni* feeding activity under ET conditions, we used tandem-mass tag proteomic analyses to investigate the effect of ET on the composition of plant and insect proteins excreted from the insect gut. Consistent with our observations that ET did not alter JA-based defenses in the host plant, Arabidopsis proteins associated with JA and wound responses accumulated to similar levels in frass collected from *T. ni* feeding at both temperatures. However, our analysis showed that the abundance of two β-thioglucoside glucohydrolase (TGG) enzymes, which hydrolyze glucosinolates into toxic derivatives in the insect gut [34], was reduced under ET conditions. These results suggest that elevated temperatures may affect the conversion of glucosinolates into toxic derivatives within the insect gut and highlight the need for future studies of how temperature modulates post-ingestive plant defenses. Nonetheless, a main finding from our study is that insect proteins (e.g., proteases) having likely roles in food digestion were enriched in frass from ET-reared insects. Together with the observation that ET conditions stimulate the rate of leaf consumption and larval growth, we suggest that elevated production of digestive enzymes may be needed to efficiently process the increased amount of leaf tissue ingested at ET. In future studies, it will be of interest to determine whether higher temperatures impact the capacity of *T. ni* to detoxify chemical defenses in the leaf diet [33,42,43]. It is possible that temperature-dependent increases in the abundance and activity of digestive enzymes reduces a bottleneck in the conversion of host tissue to insect biomass, thereby allowing faster ingestion of plant tissue. Such bottlenecks represent potential targets for the development of new insecticides to help protect crops in a warming world.

## 4. Materials and Methods

Plant materials and growth conditions: The Columbia accession (Col-0) of *A. thaliana* was used as the wild type (WT) for all the experiments. The JA-deficient mutant defective in *ALLELE OXIDE SYNTHASE* (*AOS*) was backcrossed into the Col-0 genetic background [25]. Seed for the *aos* mutant was propagated by spraying plants with a solution containing methyl-JA, as previously described [46,47]. WT and *aos* seeds were stratified in water in the dark at 4 °C. Following sowing of seeds in soil, potted plants were covered with a transparent plastic dome for approximately 10 days to facilitate seedling establishment. Soil-grown plants were maintained under CT conditions (22 °C, 8 h under 100 μE m^−2^ s^−1^ and 16-h dark photoperiod) unless otherwise noted. For video monitoring of insect behavior, plants were sown in Jiffy peat pellets.

Insect feeding assays: Insect feeding assays adapted from Herde and Howe [33] were performed at 22 °C or 29 °C under a short-day photoperiod of 8-h light and 16-h dark as described above. For insect growth assays, neonate *Trichoplusia ni* larvae (Benzon Research) were transferred to fully expanded rosette leaves of eight-week-old WT and *aos* plants previously grown under CT conditions. Before challenge, plants were acclimated to test temperatures for 48 h. Two larvae were reared on each of five plants. Larval weights were measured at various times after challenge and the weighed larvae were returned to the plants to continue feeding. Insect relative growth rate was calculated as the slope of the common log of larval weight plotted against time. Therefore, RGR was calculated from the 3rd to the 6th day of feeding for larvae reared at elevated temperature (ET) and from the 3rd to 11th day of feeding for larvae reared at the control temperature (CT). For video recording of insect feeding behavior, a Mako G-503 PoE camera was used to record insects that were reared on the WT at CT for at least seven days and then transferred to new WT or *aos* plants at the test temperature. Insects were allowed to acclimate for 24 h on these plants at the test temperature before video recording. Bite rates were estimated for feeding bouts by counting the number of bites made within a 30-sec window. A single feeding session recorded over approximately seven hours constituted one biological replicate. Slow motion was used to estimate bite rate if necessary. Statistical analysis (ANOVA, Tukey’s Honestly Significant Difference) were performed using the GraphPad Prism software suite.

Hormone quantification: Plants were grown for 38 days under CT conditions prior to transfer to CT or ET treatment chambers. Following a two-day acclimation period in the treatment chambers, three leaves were mechanically damaged across the midvein with a hemostat. JAs were extracted as previously described [48], with minor modifications. Briefly, frozen tissue was ground in a ball mill and extracted in 80:20 (v/v) methanol:water solution containing 0.1% formic acid. ^13^C6-JA-Ile and dihydro-JA were used as internal standards for quantification of JA-Ile and JA, respectively. Hormone measurements were performed using a Quattro premier LC-MS-MS (Waters), as previously described [48].

mRNA quantification. Plants were grown for 38 days prior to transfer to CT and ET treatment chambers. Following a two-day acclimation period in the treatment chambers, leaves were mechanically damaged as described above. Damaged leaves were collected at various times after wounding and immediately flash-frozen in liquid nitrogen. RNA was purified using a NucleoSpin RNA Plant extraction kit (Machery-Nagel) and cDNA was synthesized using superscript III (ThermoFisher Scientific). Marker gene transcripts were quantified using real-time quantitative PCR (RT-qPCR) with SYBR Green reagents and primers listed in Supplemental Table S3. Gene expression levels were normalized to transcripts encoding a regulatory subunit of PROTEIN PHOSPHATASE 2A (At1g13320) using the Δ ΔCt method [49].

Glucosinolate measurements: Plants were grown under CT conditions for four weeks prior to transfer to CT or ET test chambers. Following a seven-day acclimation period in the treatment chambers, rosette leaves were harvested and immediately frozen in liquid nitrogen. Two plants were pooled for each sample, with three biological replicates collected per sample. Frozen tissue was homogenized with a TissueLyser II (Qiagen) and glucosinolates were extracted following published procedures [50], with minor modifications. Briefly, 80% methanol (v/v) was added to homogenized tissues and the mixture was vortexed for five min. Extracts were then centrifuged at 16,000× *g* for five min and the supernatant was diluted 10-fold in water and transferred to a two-mL glass vial (RESTEK). Samples were analyzed in the MSU Mass Spectrometry Facility by ultrahigh pressure liquid chromatography (UPLC) coupled to quadrupole time-of-flight mass spectrometry (QTOFMS) using Waters Xevo G2-XS [50,51].

Proteomic analysis: Frass was collected from early instar caterpillars feeding on 38 day-old WT plants under CT or ET conditions. Host plants were grown continuously under the CT conditions, and caterpillars were reared on plants at CT until the third instar. Plants and insects were acclimated to test temperatures for 24 h before collecting frass into liquid nitrogen throughout the 24-h feeding trial. Frozen frass was ground in a ball mill and mixed with protein extraction buffer (0.7 M sucrose, 100 mM Tris HCL, 20 mM EDTA, 100 mM KCL, 5% 2-mercaptoethanol, 1 mM phenylmethylsulfonyl fluoride, and cOmplete mini EDTA-free protease inhibitor cocktail) as previously described [35] with minor modifications. Briefly, proteins were separated in one volume of water-saturated phenol and centrifuged for five min at 10,000× *g*. The protein-containing phenol phase was collected and precipitated in 100 mM ammonium acetate in methanol at −20 °C. Following centrifugation, the pellet was washed in 80% acetone before resuspension in 9.5 M urea, 2% noident and 5% (v/v) 2-mercaptoethanol. Protein samples were digested with trypsin using the Filter-Aided Sample Preparation (FASP) protocol according to Wisniewski et al. [52] using spin ultrafiltration units with a nominal MW cutoff of 30,000 Da. The resulting peptides were de-salted using reverse phase C18 Stage-Tips and dried by vacuum centrifugation. Samples were then labeled with 6-plex TMT reagents (www.thermo.com) according to the manufacturer’s protocol. Eluted peptides were sprayed into a ThermoFisher Q-Exactive HF-X mass spectrometer (www.thermo.com) using a FlexSpray nano-spray ion source. Survey scans were taken in the Orbi trap (120,000 resolution, determined at *m*/*z* 200) and the top ten ions in each survey scan were then subjected to automatic higher energy collision induced dissociation (HCD) with fragment spectra acquired at 45,000 resolution. Conversion of MS/MS spectra to peak lists and quantitation of TMT reporter ions was done using Proteome Discover, v2.2.0.338. Peptide-to-spectrum matching was done using the Sequest HT, Mascot and X!Tandem search algorithms against the TAIR, v10, sequence database (Arabidopsis) and the recently published *Trichoplusia ni* sequence database [36] appended with common laboratory contaminants (downloaded from www.arabidopsis.org and www.thegpm.org, respectively). The output from all three search algorithms was then combined and analyzed using Scaffold Q+S, v4.8.7 (www.proteomesoftware.com) to probabilistically validate protein identifications and quantification. Assignments validated using the Scaffold 1% FDR confidence filter were considered true. The PANTHER classification and search tool was used to search Arabidopsis protein lists for functional annotations [53].

## Supplementary Materials

The following are available online at https://www.mdpi.com/2223-7747/9/2/172/s1: Video S1, Real time video of larvae feeding on WT and *aos* plants under CT and ET conditions; Figure S1, larval feeding behavior traces; Table S1, Arabidopsis proteins identified in frass; Table S2, *T. ni* proteins identified in frass; Table S3, oligonucleotide primers used in this study.
