# Supplementary material for: Stimulation of Insect Herbivory by Elevated Temperature Outweighs Protection by the Jasmonate Pathway
Source: Plants (Basel). 2020 Feb 1;9(2):172. doi: 10.3390/plants9020172 (PMC7076421; doi:10.3390/plants9020172)
Supplement: Supplementary file 1 [file plants-09-00172-s001.zip › Supplemental Material/Supplementary File 1.pdf]

Supplementary materials for Havko et al.

Stimulation of insect herbivory by elevated temperature outweighs protection by the jasmonate pathway

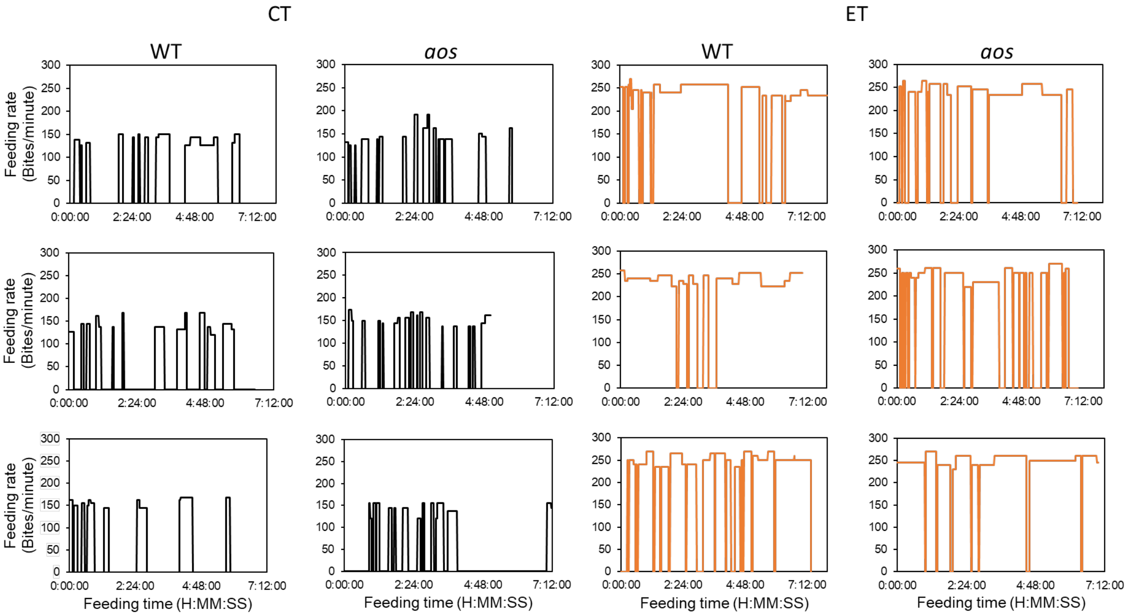

Figure S1. *T. ni* larvae spend more time feeding and feed faster at elevated temperature. WT and *aos* mutant plants were grown continuously at CT for 8 weeks and then transferred to CT or ET treatment chambers. Following a 24-h acclimation period in the treatment chamber, plants were challenged with *T. ni* larvae that had been reared on the WT for at least 7 days to a weight of approximately 35 mg. Within the treatment chambers, larvae were allowed to feed for 24-h prior to video monitoring. Data show traces of the activity of each individual larvae monitored. Slow motion was used to facilitate counts of bites when necessary.

Rank	Protein Annotation	Accession Number	Fold change ET/CT
1	Leucine-rich repeat (LRR) family protein	AT3G24480.1	3.62
2	BIP1, Heat shock protein 70 (Hsp 70) family protein	AT5G28540.1	2.75
3	CYN, Cyanase	AT3G23490.1	2.38
4	Leucine-rich repeat (LRR) family protein	AT4G18670.1	1.96
5	RD21, Granulin repeat cysteine protease family protein	AT1G47128.1	1.90
6	CSY1, Citrate synthase 1	AT3G58740.1	1.89
7	RmlC-like cupins superfamily protein	AT3G05950.1	1.79
8	Histone superfamily protein	AT1G07660.2	1.69
9	ATFER4, Ferritin 4	AT2G40300.1	1.63
10	Thioesterase superfamily protein	AT2G22230.1	1.63
11	CSD2, copper/zinc superoxide dismutase 2	AT2G28190.1	1.59
12	Myosin heavy chain-related	AT5G61200.1	1.56
13	DPP6 N-terminal domain-like protein	AT1G21680.1	1.56
14	TIP2, Tonoplast intrinsic protein 2	AT3G26520.1	1.55
15	TIP2 Tonoplast intrinsic protein	AT4G17340.1	1.54
16	ATPB, ATP synthase subunit beta	ATCG00480.1	1.50
17	VDAC1, Voltage dependent anion channel	AT3G01280.1	1.49
18	Glucose-methanol-choline oxidoreductase family protein	AT3G56060.1	1.49
19	Xanthine/uracil permease family protein	AT5G62890.1	1.49
20	LHCB2.2, photosystem II light harvesting complex	AT2G05070.1	1.49
21	ATEXLB1	AT4G17030.1	1.43
22	ATSBT3.5, Subtilase family protein	AT1G32940.1	1.42
23	Pectin lyase-like superfamily protein	AT3G57790.1	1.42
24	ATAMT2, Ammonium transporter 2	AT2G38290.1	1.38
25	Protein of unknown function (DUF567)	AT5G01750.1	1.38
26	Polynucleotidyl transferase, ribonuclease H-like superfamily protein	AT3G11770.1	1.36
27	AAO3, Abscisic aldehyde oxidase 3	AT2G27150.1	1.33
28	Protein of unknown function, DUF642	AT5G11420.1	1.32
29	RD20, Caleosin-related family protein	AT2G33380.1	1.31
30	PYR4, Pyrimidin 4	AT4G22930.1	1.30
31	Leucine-rich repeat protein kinase family protein	AT5G59680.1	1.30
32	PSBD Photosystem II reaction center protein D	ATCG00270.1	1.30
33	Zn-dependent exopeptidases superfamily protein	AT5G60160.1	1.29
34	FAD-binding berberine family protein	AT4G20860.1	1.29
35	Glyceraldehyde 3-phosphate dehydrogenase A subunit	AT3G26650.1	1.28
36	FAD-binding berberine family protein	AT1G26390.1	1.27
37	Glycosyl hydrolase family protein with chitinase insertion domain	AT4G19810.1	1.27
38	Cell wall, plant-type cell wall protein	AT1G21670.1	1.26
39	Receptor-like protein kinase-related family protein	AT5G48540.1	1.26
40	GGT1 Gamma-glutamyl transpeptidase 1	AT4G39640.1	1.24
41	BNR/Asp-box repeat family protein	AT5G57700.1	1.23
42	mMDH1, Lactate/malate dehydrogenase family protein	AT1G53240.1	1.21
43	SBPASE, Sedoheptulose-bisphosphatase	AT3G55800.1	1.21
44	DHAR1, Dehydroascorbate reductase	AT1G19570.1	1.21
45	UCC2 Uclacyanin 2	AT2G44790.1	1.21
46	HOT5	AT5G43940.1	1.21
47	FIB4, Plastid-lipid associated protein PAP / fibrillin family protein	AT3G23400.1	1.18
48	Arginase/deacetylase superfamily protein	AT4G08870.1	1.18
49	LHCB3, Light-harvesting chlorophyll B-binding protein 3	AT5G54270.1	1.18
50	GAPB, Glyceraldehyde-3-phosphate dehydrogenase B subunit	AT1G42970.1	1.16
51	LOX2, Lipoxygenase 2	AT3G45140.1	1.15
52	FAD-binding Berberine family protein	AT2G34810.1	1.15
53	SS2 Strictosidine synthase 2	AT1G74020.1	1.14
54	CARB, Carbamoyl phosphate synthetase B	AT1G29900.1	1.13
55	Peptidase M1 family protein	AT1G63770.1	1.13
56	GLP5, Germin-like protein 5	AT1G09560.1	1.13
57	UVR8, Regulator of chromosome condensation (RCC1) family protein	AT5G63860.1	1.12
58	ATP-dependent Clp protease	AT1G33360.1	1.11
59	AIR12 Auxin-responsive family protein	AT3G07390.1	1.11
60	Glycosyl hydrolase family 38 protein	AT3G26720.1	1.10
61	ATCWINV1, Glycosyl hydrolases family 32 protein	AT3G13790.1	1.10
62	LIN2, Coproporphyrinogen III oxidase	AT1G03475.1	1.10
63	GLP3, Germin 3	AT5G20630.1	1.09
64	D-mannose binding lectin protein with Apple-like carbohydrate-binding domain	AT1G78850.1	1.08
65	ARA12, Subtilase family protein	AT5G67360.1	1.05
66	Glycosyl hydrolase family 38 protein	AT5G13980.1	1.05
67	HSP60-2 Heat shock protein 60-2	AT2G33210.1	1.03
68	Alkaline-phosphatase-like family protein	AT4G29680.1	1.03
69	Ribosomal protein S27a	AT1G23410.1	1.03
70	Polyketide cyclase/dehydrase and lipid transport superfamily protein	AT4G23670.1	1.03
71	PSBB, Photosystem II reaction center protein B	ATCG00680.1	1.01
72	Receptor-like protein kinase-related family protein	AT3G22060.1	1.01
73	FAD-binding berberine family protein	AT5G44400.1	1.01
74	ATTIL, Temperature-induced lipocalin	AT5G58070.1	1.00
75	ARO1, Armadillo repeat only 1	AT4G34940.1	1.00
76	PSAD-2, Photosystem I subunit D-2	AT1G03130.1	1.00
77	TRAF-like family protein	AT1G58270.1	1.00
78	Lipase/lipooxygenase, PLAT/LH2 family protein	AT4G39730.1	1.00
79	FAD-binding berberine family protein	AT1G30700.1	1.00
80	ATGR2, glutathione reductase	AT3G54660.1	0.99
81	LHCB5, light harvesting complex of photosystem II	AT4G10340.1	0.99
82	GTP binding elongation factor Tu family protein	AT4G02930.1	0.98
83	Eukaryotic aspartyl protease family protein	AT1G03220.1	0.96
84	Glycosyl hydrolases family 32 protein	AT1G62660.1	0.96
85	MD-2-related lipid recognition domain-containing protein	AT5G23820.1	0.96
86	DRT112, Cupredoxin superfamily protein	AT1G20340.1	0.95
87	Galactose mutarotase-like superfamily protein	AT3G47800.1	0.94
88	CCD1, carotenoid cleavage dioxygenase 1	AT3G63520.1	0.93
89	CSD1, copper/zinc superoxide dismutase 1	AT1G08830.1	0.93
90	PSAA, Photosystem I, PsaA/PsaB protein	ATCG00350.1	0.93
91	HAD superfamily, subfamily IIIB acid phosphatase	AT5G44020.1	0.92
92	Alpha/beta-Hydrolases superfamily protein	AT1G27480.1	0.92
93	Serine protease inhibitor (SERPIN) family protein	AT1G47710.1	0.91
94	HAD superfamily, subfamily IIIB acid phosphatase	AT1G04040.1	0.90
95	ATTLL1, Triacylglycerol lipase-like 1	AT1G45201.1	0.89
96	TGD2 Trigalactosyldiacylglycerol2	AT3G20320.1	0.89
97	Peroxidase superfamily protein	AT1G71695.1	0.88
98	IPP2, Isopentenyl pyrophosphate	AT3G02780.1	0.87
99	VSP1, Vegetative storage protein 1	AT5G24780.1	0.87
100	Cytosol aminopeptidase family protein	AT4G30920.1	0.86
101	AOC1, Allene oxide cyclase 1	AT3G25760.1	0.86
102	Leucine-rich repeat (LRR) family protein	AT1G33600.1	0.85
103	Zn-dependent exopeptidases superfamily protein	AT5G04710.1	0.85
104	PSAB, Photosystem I, PsaA/PsaB protein	ATCG00340.1	0.85
105	ATCLH1, Chlorophyllase 1	AT1G19670.1	0.83
106	Curculin-like lectin family protein	AT1G78830.1	0.82
107	ATPME3, Pectin methylesterase 3	AT3G14310.1	0.82
108	MD-2-related lipid recognition domain-containing protein	AT3G44100.1	0.81
109	PERX34, Peroxidase CB	AT3G49120.1	0.80
110	Ribulose-bisphosphate carboxylases	ATCG00490.1	0.80
111	Subtilisin-like serine endopeptidase family protein	AT2G04160.1	0.79
112	Dehydratase family	AT3G23940.1	0.78
113	FAD-binding berberine family protein	AT4G20830.1	0.76
114	Dihydrolipoyl dehydrogenases	AT4G16155.1	0.76
115	Subtilase family protein	AT3G14067.1	0.75
116	Cytosol aminopeptidase family protein	AT2G24200.1	0.74
117	JAL23, Jacalin-related lectin 23	AT2G39330.1	0.73
118	PETA, Photosynthetic electron transfer A	ATCG00540.1	0.72
119	PIP2B, Plasma membrane intrinsic protein 2	AT2G37170.1	0.72
120	CORI3, Tyrosine transaminase family protein	AT4G23600.1	0.72
121	NIR1, Nitrite reductase 1	AT2G15620.1	0.71
122	WIN1, HOPW1-1-interacting 1	AT1G80600.1	0.71
123	TRAF-like family protein	AT3G28220.1	0.70
124	AGT, Alanine glyoxylate aminotransferase	AT2G13360.1	0.69
125	Xylose isomerase family protein	AT5G57655.2	0.69
126	GSO1 | Leucine-rich repeat transmembrane protein kinase	AT4G20140.1	0.68
127	Galactose mutarotase-like superfamily protein	AT4G25900.1	0.66
128	PSBC, Photosystem II reaction center protein C	ATCG00280.1	0.65
129	Leucine-rich repeat (LRR) family protein	AT1G49750.1	0.65
130	TGG2, Glucoside glucohydrolase 2	AT5G25980.2	0.65
131	Leucine-rich repeat (LRR) family protein	AT1G33590.1	0.62
132	Eukaryotic aspartyl protease family protein	AT3G52500.1	0.61
133	CAT3, Catalase 3	AT1G20620.1	0.59
134	MD-2-related lipid recognition domain-containing protein	AT3G11780.1	0.50
135	PETE1 Plastocyanin 1	AT1G76100.1	0.43
136	TGG1, Thioglucoside glucohydrolase 1	AT5G26000.1	0.43

Table S1. Arabidopsis proteins in frass from *T. ni* larvae reared at control versus elevated temperature. *T. ni* larvae were reared on WT Arabidopsis plants under CT or ET conditions as described in the main text. Arabidopsis proteins excreted in caterpillar frass (feces) were identified and quantified using a tandem-mass tag shotgun proteomics approach (see Methods). Listed are all Arabidopsis proteins showing increased (red) or decreased (green) abundance under ET compared to CT conditions.

Rank	Protein annotation	Accession Number	Fold change (ET/CT)
1	Lipase 3-like	XP_026730889.1	6.83
2	Uncharacterized protein	XP_026735425.1	4.20
3	Xanthine dehydrogenase-like	XP_026741596.1	3.05
4	Alpha-amylase 1-like	XP_026735018.1	2.86
5	Uncharacterized protein	XP_026741374.1	2.60
6	Glucose dehydrogenase [FAD, quinone]-like	XP_026725148.1	2.56
7	Trypsin CFT-1-like	XP_026736003.1	2.49
8	Collagenase-like	XP_026730456.1	2.32
9	Lipase member H-like	XP_026747853.1	2.25
10	Collagenase-like	XP_026730856.1	2.19
11	Dihydrolipoyllysine-residue succinyltransferase	XP_026734712.1	2.17
12	ATP synthase subunit alpha, mitochondrial	XP_026741163.1	2.08
13	Uncharacterized protein	XP_026728305.1	2.06
14	Trypsin, alkaline C-like	XP_026732818.1	1.96
15	CDK5RAP3-like protein	XP_026727222.1	1.92
16	Inactive pancreatic lipase-related protein 1-like	XP_026731478.1	1.86
17	Collagenase-like	XP_026730522.1	1.85
18	ATP synthase subunit beta, mitochondrial-like	XP_026737421.1	1.80
19	Trypsin, alkaline C-like	XP_026733377.1	1.79
20	Alpha-amylase 2-like	XP_026741915.1	1.74
21	Pancreatic triacylglycerol lipase-like	XP_026733233.1	1.69
22	Uncharacterized protein	XP_026732755.1	1.67
23	Uncharacterized protein	XP_026730703.1	1.64
24	Trypsin, alkaline C-like	XP_026739088.1	1.63
25	Uncharacterized protein	XP_026726201.1	1.63
26	Pancreatic triacylglycerol lipase-like	XP_026731477.1	1.62
27	Zinc carboxypeptidase-like	XP_026743018.1	1.59
28	Collagenase-like isoform X1	XP_026730214.1	1.59
29	Uncharacterized protein	XP_026728307.1	1.57
30	Midgut glutamate specific carboxypeptidase, partial	AAS82585.1 (+1)	1.56
31	Collagenase-like isoform X1	XP_026730441.1	1.56
32	Trypsin CFT-1-like	XP_026739086.1	1.55
33	Uncharacterized protein	XP_026726072.1	1.54
34	Cadherin	AEA29692.1 (+1)	1.53
35	Uncharacterized protein	XP_026726462.1	1.50
36	Uncharacterized protein	XP_026740026.1	1.49
37	Pancreatic lipase-related protein 2-like	XP_026747854.1	1.46
38	PiggyBac transposable element-derived protein 3-like	XP_026746698.1	1.46
39	Collagenase-like	XP_026728548.1	1.45
40	Venom dipeptidyl peptidase 4-like isoform X1	XP_026746174.1	1.45
41	Uncharacterized protein	XP_026733613.1	1.45
42	Alkaline phosphatase-like	XP_026741480.1	1.45
43	Beta-1,3-glucan-binding protein-like	XP_026729328.1	1.43
44	Trypsin CFT-1-like isoform X1	XP_026739083.1	1.42
45	Pancreatic lipase-related protein 2-like	XP_026731490.1	1.40
46	Brachyurin-like	XP_026727644.1	1.39
47	Trypsin, alkaline B-like	XP_026733144.1	1.39
48	Aldehyde oxidase 1-like	XP_026741880.1	1.36
49	Alkaline phosphatase	AEG79734.1	1.36
50	Trypsin, alkaline B-like	XP_026739095.1	1.36
51	Uncharacterized protein	XP_026735424.1	1.35
52	Trypsin, alkaline B-like	XP_026739094.1	1.33
53	Collagenase-like	XP_026730235.1	1.31
54	Azurocidin-like precursor protein	AAM46727.1	1.30
55	Carboxypeptidase B-like	XP_026736474.1	1.30
56	Uncharacterized protein	XP_026725494.1	1.29
57	Pancreatic triacylglycerol lipase-like	XP_026731590.1	1.27
58	Chymotrypsin-1-like	XP_026741849.1	1.25
59	Glucosylceramidase-like	XP_026747406.1	1.25
60	Lipase member H-like	XP_026731597.1	1.24
61	Apoptotic chromatin condensation inducer in the nucleus-like	XP_026738550.1	1.24
62	Zinc carboxypeptidase-like	XP_026743124.1	1.24
63	Chymotrypsin-2-like	XP_026741847.1	1.23
64	Pancreatic lipase-related protein 2-like	XP_026747862.1	1.23
65	Midgut carboxypeptidase 1, partial	AAS82586.1 (+2)	1.23
66	Uncharacterized protein	XP_026726105.1	1.22
67	Brachyurin-like	XP_026731127.1	1.21
68	Renin receptor	XP_026731297.1	1.21
69	Pancreatic lipase-related protein 2-like	XP_026731672.1	1.21
70	Apyrase-like	XP_026728055.1	1.18
71	Transmembrane protease serine 9-like	XP_026730702.1	1.18
72	Cluster of xanthine dehydrogenase-like	XP_026741695.1	1.18
73	Lipase 3-like	XP_026725377.1	1.15
74	Vanin-like protein 2 isoform X2	XP_026740921.1	1.13
75	Lipase 3-like	XP_026730913.1	1.10
76	Pancreatic lipase-related protein 2-like	XP_026747863.1	1.09
77	Uncharacterized protein	XP_026743093.1	1.08
78	Sphingomyelin phosphodiesterase 1-like	XP_026745062.1	1.08
79	Chymotrypsin-1-like	XP_026741845.1	1.05
80	Serine protease 1-like	XP_026746126.1	1.04
81	Cluster of lipase 1-like	XP_026730901.1	1.02
82	Trypsin, alkaline C-like	XP_026736002.1	1.01
83	Uncharacterized protein	XP_026738773.1	1.00
84	Pancreatic lipase-related protein 2-like	XP_026740983.1	1.00
85	Uncharacterized protein	XP_026726465.1	0.96
86	Achelase-1-like	XP_026739079.1	0.96
87	XAA-Pro aminopeptidase ApepP-like	XP_026747928.1	0.96
88	patj homolog	XP_026743074.1	0.95
89	Lipase 3-like	XP_026739037.1	0.93
90	Pancreatic triacylglycerol lipase-like	XP_026740985.1	0.93
91	Trypsin, alkaline C-like	XP_026739087.1	0.89
92	Aminopeptidase N3	AAX39865.1 (+1)	0.89
93	Glucose dehydrogenase [FAD, quinone]-like	XP_026740327.1	0.88
94	Esterase B1-like	XP_026744596.1	0.87
95	Histone H2B	XP_026746532.1	0.84
96	Membrane alanyl aminopeptidase-like	XP_026737522.1	0.83
97	Uncharacterized protein	XP_026737525.1	0.83
98	Lipase member I-like	XP_026733234.1	0.82
99	Gamma-glutamyl hydrolase A-like	XP_026731422.1	0.77
100	Uncharacterized protein	XP_026734246.1	0.76
101	Aminopeptidase N2	AAX39864.1 (+1)	0.74
102	Uncharacterized protein	XP_026726460.1	0.74
103	Cluster of esterase FE4-like	XP_026747801.1	0.73
104	Collagenase-like	XP_026730342.1	0.68
105	Carboxypeptidase B-like	XP_026736537.1	0.68
106	Uncharacterized protein	XP_026734361.1	0.62
107	Uncharacterized protein	XP_026730682.1	0.55
108	Glucose dehydrogenase [FAD, quinone]-like	XP_026735683.1	0.31

Table S2. *Trichoplusia ni* proteins in frass from larvae reared at control versus elevated temperature. *T. ni* proteins excreted in caterpillar frass (feces) were identified and quantified using a tandem-mass tag shotgun proteomics approach (see Methods). Listed are all *T. ni* proteins showing increased (red) or decreased (green) abundance under ET compared to CT conditions.

Table S3. Oligonucleotide primers used in this study.

Gene	AGI ID	Primer name	Sequence (5'-3')
VEGETATIVE STORAGE PROTEIN2	AT5G24770	VSP2F	GTTAGGGACCGGAGCATCAACC
		VSP2R	TCTTCCACAACTTCCAACGGTCACT
LIPOXYGENASE 3	AT1G17420	LOX3F	GCTGGCGGTTCGACATG
		LOX3R	GCCATTCCTCTGCGAATTAGA
PROTEIN PHOSPHATASE 2A	AT1G13320	PP2AF	AAGCAGCGTAATCGGTAGG
		PP2AR	GCACAGCAATCGGGTATAAAG
